# Attitudes Towards Assisted Dying in Dementia: A Focus Group Study with Younger and Older Adults

**DOI:** 10.1177/00302228211063297

**Published:** 2021-12-07

**Authors:** Freya Thompson, Alexandra R. Nelson, Rachel O. Coats, Judith Johnson

**Affiliations:** 1 School of Psychology, 4468 University of Leeds, Leeds, UK; 2 Department of Psychology, 3158 University of Alberta, Edmonton, AB, Canada; 3 Bradford Institute for Health Research, Bradford Royal Infirmary, Bradford, UK; 4 School of Public Health and Community Medicine, University of New South Wales, Sydney, NSW, Australia

**Keywords:** dementia, assisted dying, euthanasia

## Abstract

**Objectives**: To explore attitudes towards assisted dying in dementia (ADID) and the rationales underlying these attitudes, among younger and older adults.

**Method**: We conducted separate focus groups with younger (*n* = 11) and older adults (*n* = 14) in the United Kingdom with personal or professional experience of dementia. Discussions were prompted by two vignettes depicting scenarios of ADID. The data were transcribed and analysed using thematic analysis.

**Results**: Though sometimes stronger in the older adults, many of the attitudes and underlying rationales were common across the age groups. Analysis generated four themes: ‘Perceptions of the disease’, ‘A case for empowerment’, ‘The morality of killing’ and ‘Logistical complexities’.

**Conclusions**: For some, ADID was a hopeful alternative to the challenges they had witnessed in dementia. For others, the logistical problems surrounding ADID were insurmountable. Discussions were informed and insightful, highlighting the importance of including the general public in this ongoing debate.

## Introduction

By 2050, an estimated one in four people in Europe will be aged 65 or over, compared with just one in 11 at present (United Nations Department of Economic and Social Affairs P. D., 2020, 2019). Through this demographic shift, the prevalence of age-related degenerative diseases such as dementia will increase dramatically, with a projected rise from 50 million to 151 million dementia cases worldwide by 2050 (Prince et al., 2014). The rising prevalence of dementia presents significant challenges for the individual and society; dementia is the most feared illness in over 65s (Richards, 2017) and the yearly public costs by 2040 are estimated at £55bn in the United Kingdom alone (Prince et al., 2014). With no immediate cure in sight, discussions are increasingly turning to the question of legalising assisted dying in dementia (ADID; Menzel & Steinbock, 2013; Tomlinson & Stott, 2015).

The term assisted dying encompasses both euthanasia, in which a doctor administers a patient with a lethal drug to terminate their life, and physician-assisted dying, where a patient is provided with a lethal drug for self-administration (Materstvedt et al., 2003). Countries where assisted dying is legal have strict safeguards to ensure the safety and security of both the patient and the doctors involved. Typically, these safeguards limit assistance to terminally ill adults who are suffering unbearably and are deemed to be competent to make a voluntary request to die (Kouwenhoven et al., 2015). In the majority of jurisdictions where assisted dying is legal, it is not permitted for individuals with dementia, as the progressive functional decline characteristic of the disease affects mental competence and decision-making ability (De Boer et al., 2011).

In many countries where the practice is not legal, legalising ADID receives significant public support, though the issue is often highly polarised (Terkamo-Moisio et al., 2019; Williams et al., 2007). Understanding the attitudes of the public is essential, yet a paucity of literature exists and the views of adults under 50 are rarely included (Cox et al., 2013; Hendry et al., 2013). This gap is significant as any change in the law on a controversial issue is typically preceded by a public referendum (Mroz et al., 2020). Developing the debate and informing policymakers of the issues pertinent to the public is, therefore, crucial (Hendry et al., 2013). Furthermore, in-depth qualitative analysis has been recommended for studying this complex debate, as survey data has been shown to be an inadequate methodology (Cox et al., 2013; Magelssen et al., 2016). Finally, it is younger adults who face a future of greater longevity and related health burdens so their perspectives must be included (Cox et al., 2013).

To address these gaps in the literature, we conducted a qualitative study that aimed to explore attitudes towards ADID and the rationales underlying these attitudes. We included both younger and older adults with personal or professional experience of dementia. The study was conducted in the United Kingdom, a jurisdiction where physician-assisted death and euthanasia are currently illegal in any situation.

## Methods

### Approach

We took a phenomenological approach, considered within an interpretivist paradigm and analysed the data using thematic analysis (Braun & Clarke, 2006). Our theoretical approach was based on attitude formation theory, specifically the tripartite model of attitudes (Rosenberg & Hovland, 1960). According to the tripartite model, the internal structure of an attitude consists of three components: the affective component (an individual’s emotional response to a subject), the cognitive component (their thoughts and beliefs towards the subject) and the behavioural component (their behavioural intentions around the subject; Kaiser & Wilson, 2019). The three components are relied on differently depending on the nature of the attitude object, the personality of the individual and the amount of experience relating to the attitude object (Ajzen, 2001). We used this model to inform our focus group discussion guide and to facilitate a more in-depth interpretation of the data.

### Participants and Recruitment

Participants were 14 older adults (aged 60 and over) and 11 younger adults (aged 18–25 years), recruited via volunteer sampling methods (Table 1). One younger adult dropped out due to sickness on the day of the focus group. We recruited younger adults through social media and posters around a university in the North of England. Older adults were recruited from a university participant pool and responded to an email if they wished to take part. To promote some consistency in experience amongst participants, we recruited on the basis of having some experience of dementia in either a personal or professional capacity. Personal experience was specified as experience with a relative or friend with dementia and professional experience was specified as having worked directly with individuals with dementia.

**Table 1.** Focus Group Characteristics.

Focus Group (FG)	FG1, *Older adult (n = 5)*	FG2, *Older adult* (*n* = 5)	FG 3, *Older adult* (*n* = 4)	FG4, *Younger adult* (*n* = 7)	FG 5, *Younger adult* (*n* = 5)
Characteristics	n (%)	n (%)	n (%)	n (%)	n (%)
Gender					
Female	4 (80)	5 (100)	3 (75)	4 (57)	4 (80)
Male^a^	1 (20)	0 (0)	1 (25)	3 (43)	1 (20)
Age (years)					
18–25	0 (0)	0 (0)	0 (0)	7 (100)	5 (100)
60–70	2 (40)	3 (60)	4 (100)	0 (0)	0 (0)
70–85	3 (60)	2 (40)	0 (0)	0 (0)	0 (0)
Education					
Sixth form/college	2 (40)	1 (20)	2 (50)	0 (0)	0 (0)
Higher education	3 (60)	4 (80)	2 (50)	7 (100)	5 (0)
Religion					
No religion	1 (20)	3 (60)	0 (0)	7 (100)	4 (80)
Christianity^b^	4 (80)	2 (40)	4 (100)	0 (0)	1 (20)
Marital status					
Single	0 (0)	0 (0)	0 (0)	7 (0)	5 (0)
Married	3 (60)	1 (20)	2 (50)	0 (0)	0 (0)
Widowed	1 (20)	2 (40)	0 (0)	0 (0)	0 (0)
Divorced	1 (20)	2 (40)	2 (50)	0 (0)	0 (0)

^a^ Only two genders reported in the sample.

^b^ Christianity was the only religion reported in the sample and includes various denominations.

Recruitment was ceased once an appropriate sample size was reached, with consideration of obtaining sufficient ‘information power’ (Malterud et al., 2016). The number of participants required to achieve sufficient information power depends on the study aims, sample specificity, theoretical background, quality of the dialogue and method of analysis (Malterud et al., 2016). We appraised information power periodically throughout the design and recruitment process and whilst the study was of an exploratory nature, the homogeneity of the sample and length and depth of discussions meant that by the final two focus groups, initial themes were being reinforced rather than changed or increased in number and the researchers agreed a sufficient sample size had been reached.

### Data Collection

The focus groups took place at the university campus between February and March 2020; 3 with older adults and 2 with younger adults, each lasting on average 90 minutes. We separated the groups by age in order to compare attitudes between age groups. Within each age group participants were allocated to a focus group pragmatically, according to the date and time that best suited their availability. Each group had between four and seven participants; the size was chosen to enable fluid discussions between participants but not so large as to discourage disclosure of personal experience (Gill et al., 2008). Written informed consent was obtained from each participant before the focus group and discussions were audio-recorded, transcribed and anonymised by the first author (FT). Prior to the discussions, participants were asked to fill in a short demographics form. Ethical approval was gained from the University’s Ethics Committee on February 13, 2020, reference number (PSC-910).

### Focus Group Discussions

**Figure 1.**
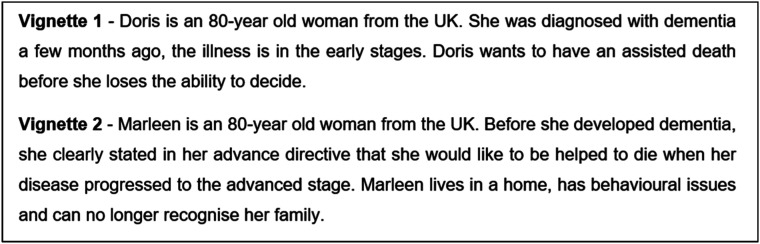
Vignettes used in the study.

**Figure 2.**
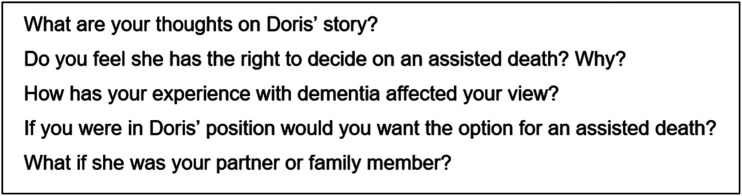
Example prompts for vignette 1.

All authors developed a flexible discussion guide that allowed space for the participants to share their experiences and insights. Focus groups were conducted by FT, with field notes taken concurrently by AN. Discussions began with a warm-up card sort task designed to encourage participants to reflect on what values are important to them at the end of life (Colucci, 2007). Next, discussions focused on two separate vignettes depicting 80-year-old females: one in the early stages of dementia and one in the later stages (Figure. 1). Participants were asked to reflect on each case, with the use of prompts to stimulate conversation (Figure. 2).

### Data Analysis

We used NVivo 12 software to facilitate data coding. Focus groups were analysed using thematic analysis (Braun & Clarke, 2006). FT transcribed all five transcripts, at the same time becoming familiar with the data and generating initial codes. Additionally, two of the transcripts were independently coded by RC and JJ to enhance the credibility and trustworthiness of the findings. All authors then met to triangulate views, discuss interpretation and contribute to theme development. Themes were derived from the data and repeated revisions were made until final themes were decided upon. The research team members had professional experience of working with dementia (FT, AN and JJ), and one had personal lived experience (AN). Team members were divided in their views on ADID, with three members tending to have more favourable attitudes (FT, AN and RC) and one member not in favour (JJ). This experience influenced the team’s interpretations of the data.

## Results

### Focus Group Characteristics

A total of 26 participants took part in the study. All participants who volunteered were of White ethnicity and Christianity was the only religion represented in the sample. Specific characteristics for each focus group are presented in Table 1.

### Themes

We developed four themes describing the data in relation to attitudes on assisted dying in dementia: ‘*Perceptions of the disease’, ‘A case for empowerment’, ‘The immorality of killing’ and ‘Logistical complexities’.* These themes were common to both the younger and older adults but varied in emphasis between the two groups.

#### Perceptions of the disease

Participants shared diverse experiences of dementia, primarily from personal relationships with parents, spouses or grandparents. These experiences shaped individual perceptions of what living and dying with dementia entailed, which in turn motivated participants’ attitudes towards assisted dying.

#### Losses from the disease

Both the younger and older adults perceived dementia as a disease causing a loss of not only memory but also personhood, dignity and quality of life. Participants described a complete loss of the person they formerly knew, which was a profound source of sadness.

It’s like you’re living with a husk of a person […] he looked like the same man obviously but the perks and the humour and the family man element was all gone (Younger adult, FG4).

Some described challenging behavioural changes which made the person with dementia’s quality of life very low:

She was a very mild gentle little woman, and she became just this terrible kind of psychotic raging little thing […] she was black and blue; she was throwing cups of tea in people’s faces (Older adult, FG2).

However, not all functional losses were regarded as detrimental to well-being. Some participants had witnessed a loss of awareness in people with dementia and this was seen to maintain or even improve quality of life.

My grandma is really far along and basically doesn’t know what’s going on most of the time and I think since reaching that point, her quality of life mentally has probably gone up (Younger adult, FG5).

#### Becoming a burden

Dementia was perceived as a disease causing a significant burden of care for family members supporting the individual with dementia. For the older adults, not burdening their families was a central driver behind supporting assisted dying. This stemmed from their own experiences of caring for their spouse or parent with dementia:

*I don’t want to be a burden on my family […] she was becoming a problem and I don’t want to become a problem with my family* (Older adult, FG1).

In contrast, the younger adults had a more accepting view of being a burden, with many feeling it was an inevitable part of ageing.

*I mean dealing with my grandparents now, it’s okay that they are sometimes a burden on us, I don’t mind being a burden for my family when the time came* (Younger adult, FG5).

An interesting exception to this was a young adult who had experienced a parent die with dementia:

*I’m sure my family wouldn’t feel burdened […] it’s more a principle that I know what their life would be like caring for me and I wouldn’t want to leave anybody that way* (Younger adult, FG4).

#### Death as Relief

Participants witnessed a long and difficult progression of dementia in their relative, some considering it the death of the person long before the individual actually died. Consequently, the eventual death of their relative felt like a relief as it released the individual from their suffering.

*I felt that the mother I knew had died long ago, and it was almost a relief* (Older adult, FG1).

Avoiding this protracted suffering was a key rationale for allowing assisted dying and some even described themselves or another family member actively wanting the person to die:

*We wanted to smother her, my sister was crying, I want her to die, I want her to die. It was like you were saying, like an animal, you wouldn’t see your little cat suffer like that* (Older adult, FG2).

Some participants made the explicit connection between their personal experience and their attitudes towards their own death and support of ADID.

*I think I might be quite biased just because I’ve watched a few people that I’ve loved die and they’ve all been quite drawn out, so I’d like just to kind of wake up and go* (Younger adult, FG4).

#### A case for empowerment

The option for an assisted death was seen as a way to gain an element of control over an otherwise uncontrollable disease.

*In dementia when you do get quite bad, you just don’t have any control physically or mentally […] So, I guess if you have some kind of assisted dying then you would still have some element of control* (Younger adult, FG4).

The older adults held particularly strong attitudes towards how they wanted to end their life.

*I certainly don’t want to be sitting in a home not knowing who I am, who they are […] I’d rather somebody put an end to it* (Older adult, FG2).

Participants valued the role ADID could play in improving end-of-life choices in dementia. Some saw ADID as an escape route that would not necessarily be used but would create peace of mind for individuals with dementia.

*It would be a comfort […] that there is a dignified death before the police are sent out after you because you’ve wandered onto the motorway in the middle of the night or something* (Older adult, FG3).

#### The Morality of Killing

Moral questions were raised regarding the implications for the doctor and the role of religion in opposing ADID.

#### Implications for the doctor

Participants questioned whether doctors would be too focused on saving lives to consider facilitating an assisted death:

*The doctors might not be so willing because it is taking a life and people haven’t really managed to unpick that from the idea of murder* (Younger adult, FG4).

Some older adults discussed end-of-life medical approaches from the 20^th^ century which were akin to assisted dying without the label. They perceived this as a humane and peaceful hastening of death and thought that the current system is too restrictive:

*In the 50s and 60s when I was a student nurse, they did not involve the relatives. The doctor decided and the number of patients that food was withheld, and they were just given water and that was the pathway that they would go* (Older adult, FG2).

*There is plenty of talk in the past that doctors increased morphine levels when they felt the patient was beyond help* (Older adult, FG1).

#### Religion and Culture

Though some of the participants held religious beliefs, none used their religious views to argue against assisted dying. Religion was raised in regard to society’s relationship with death, particularly purposeful killing:

*I think the religious element can’t really be unpicked because people associate it with killing or the implications for your soul* (Younger adult, FG4).

Some felt religion was becoming less influential on society, particularly with regard to social issues:

*There’s a lot more people that, despite the Bible saying it’s against homosexuality and abortion, are just like ‘love everyone’. Religion doesn’t dictate the law very much anymore* (Younger adult, FG5).

There was a notion, however, that broader cultural attitudes around death and dying continued to influence support for assisted dying. The idea of Western culture being avoidant of death was raised:

*I think a lot about our culture as being very death-denying […] keeping life going as long as possible comes out of our dread and terror of being dead* (Younger adult, FG4).

In contrast, some older participants thought death is now discussed more openly than it was by earlier generations. It was suggested that the discussion was not as necessary then, when problems related to living too long were less common:

*Fathers’ father was a miner, he died of lung disease and his mother died of a stroke. They never had to do any of this with their parents, never had to care for them or worry* (Older adult, FG2).

#### Logistical Complexities

Through discussing the logistics of legalising ADID, those with more ambivalent attitudes increasingly concluded that, logistically, it would not be viable. For those with strongly favourable attitudes, however, logistical problems that arose were less significant and more easily overcome.

##### A question of when

A key complexity discussed related to *when* the individual would choose to die. The early stages of the disease were seen as too early, taking away from possible ‘good times’ before the person’s disease progressed. The younger adults, in particular, shared stories of important memories and quality time they spent with their grandparents after their diagnosis:

*Maybe from more of a selfish point of view and my own experiences […] we all got to appreciate the time we had with her even though she was existing rather than living* (Younger adult, FG5).

Choosing ADID in the later stages of the disease also raised potential problems. For some of the younger adults, the need for capacity to make a voluntary decision at the time of death was paramount, especially in a disease where communicating with the person is difficult:

*It might be necessary to have the person dying while they are able to choose it voluntarily. If you wait until this [later] stage you can imagine wrestling someone into the bed to inject them and they are screaming for their mother* (Younger adult, FG4).

When discussing the possibility of an advanced directive for dementia, both age groups questioned whether individuals could make decisions for their future selves. However, this was compared to other legal documents people make at earlier stages of their life which are considered legally binding:

*I think there’s quite a lot of similarities with do not resuscitate DNR, cos you can put a DNR in 30, 40 years ago and they would still keep to that* (Younger adult, FG4).

##### A collective decision

Within every focus group, participants suggested an agreed threshold where ADID would happen. Some thought that it would have to be a collective decision between the individual, health professionals and families.

*I imagine it would be a joint decision between the families and professionals […] I think there should be a consensus reached to ensure that a particular list, the essentials, and desirables, are being achieved* (Older adult, FG3).

Several key logistical complexities arose in discussing this collective decision. Some felt that leaving the decision to health professionals and families could create undue pressure.

*Unfortunately, now it has developed she no longer has the ability to decide, now there’s pressure on the doctors, it’s confusing and stressful and it’s a lot of pressure on a lot of people* (Younger adult, FG4).

One younger adult had personal experience of her mother deciding she would want an assisted death if she were to get dementia. Though she was in favour of assisted dying in theory, in practice she found this request from her own mother distressing:

*I think I would be quite heartbroken, there’s every chance she will get it, […] there’s no way I would help her with it [assisted dying] because I would be robbed of that time myself* (Younger adult, FG4).

Some raised the potential for abuse if families were responsible for making key decisions for the individual. Others worried family members would be left feeling guilty if they facilitated their family member’s death:

*They want to prolong the agony because they can’t bear the guilt of letting it happen, families are weird aren’t they* (Older adult, FG1).

## Discussion

Younger and older adults reported both positive and negative attitudes towards legalising ADID. A consistent finding, reflected in the theme ‘*Perceptions of the disease*’, was personal lived experience being a key motivation driving these attitudes, with more negative experiences leading participants to be more in favour of legalising ADID. The theme ‘*A case for empowerment*’ reflected the possibilities for ADID to increase an individual’s sense of control, reducing their anxiety around living and dying with dementia. However, there were concerns about the morality of legalising state-endorsed deaths, particularly in terms of the impact on the doctor and religion’s relationship with death, discussed in the theme ‘*The morality of killing*’. Considered in the theme ‘*Logistical complexities*’, participants raised potential problems arising if ADID was legalised, including issues of capacity to consent, and deciding when would be the right time to die. Discussions were in-depth and insightful, with important implications for the debate.

### An Alternative Ending in Dementia Is Needed

ADID was perceived by some as a much-needed alternative to the slow and deeply saddening death from dementia they had witnessed in their relatives. Some of the older adults, in particular, had experienced significant caregiver burden, a burden they were intent on not creating for their own children. This finding raises the question of whether improving the lived experience of the individual with dementia and their family, could reduce the need/desire for ADID altogether. Indeed, end-of-life care for people with dementia has been shown to be inadequate (Dening et al., 2013; Jones et al., 2019). Issues identified include a lack of recognition of dementia as a terminal illness, limited access to end-of-life care planning and suboptimal symptom management (Lawrence et al., 2011; Martinsson et al., 2018; Poole et al., 2018). Some of the experiences shared in the present study could be mitigated by earlier and more proactive palliative intervention to support the individual with dementia and their families (Dickinson et al., 2013).

### Assisted Dying for Dementia Is Uniquely Challenging

The potential logistical problems in facilitating ADID were, for some, insurmountable. Issues of mental capacity, deciding when exactly a person would die and implications for the doctor were raised. These problems draw interesting similarities to practical problems identified in the Netherlands (Kim et al., 2020), where, euthanasia in the later stages of dementia is legal through the use of an advanced euthanasia directive (AED) (Schuurmans et al., 2019). However, the use of AEDs is limited and of the 162 people with dementia who died by assistance in 2019, only two were through an AED (Reigional Euthanasia Review Committees, 2020). A number of reasons for this have been identified, including problems meeting the criteria of due care, differing opinions between relatives and healthcare professionals and difficulties engaging in meaningful conversation with the patient around when exactly they want to die (De Boer et al., 2011; Kouwenhoven et al., 2015; Schuurmans et al., 2019). The presence of these problems in a country where the practice is legal shows the unique challenges of ADID.

Religion and the moral structures it has created around the sanctity of life and the morality of killing were seen by some participants to be a key challenge to legalising assisted dying more generally. This idea is supported in the literature, with a recent analysis of the British Social Attitudes Survey between 1983 and 2012 finding religiosity (measured by religious service attendance) to be the main influence on attitudes towards assisted dying over time (Danyliv & O’Neill, 2015). Interestingly, the adults in Focus Group 3 made the strongest statements in support of assisted dying despite all four of them reporting a religious affiliation. It is possible that this was due to our collection of information around religious affiliation rather than religiosity per se; if we had instead included a question regarding religious service attendance or another indicator of religiosity, we may have drawn different results.

### The Nature of Dementia Challenges the Role of Personal Autonomy

Respecting an individual’s autonomy in deciding when they die was another central motivation behind participants’ support for ADID. However, some key issues with this notion of autonomy surfaced. Once a person no longer has decision-making capacity, they necessarily rely on relatives and health professionals to fulfil their wishes. Subsequently, the individual’s personal autonomy is challenged as they are now subject to the will of others with their own attitudes and opinions around what is best. For instance, in our study, some of the younger adults did not want their relatives to have an assisted death and would therefore not have supported them with this. Similarly, previous research indicates doctors may not be willing to act without contemporaneous consent from the individual and may therefore reject a euthanasia request (Schuurmans et al., 2019).

To overcome these problems, some researchers and clinicians are promoting an emerging ethical concept of relational autonomy (Gómez-vírseda et al., 2019), which considers autonomy in relation to the wider system of care providers, care receivers and relatives (Gastmans & De Lepeleire, 2010). Consequently, decision-making is explicitly collective, with doctors taking an active role in deciding on the best care for patients (Walter & Ross, 2014). This new ethical focus could help bridge some of the problems arising in the Dutch system, making ADID a more viable option.

### A Theoretical Perspective Can Offer Further Insights

The focus groups revealed a key difference in attitudes, not between age groups as might be expected, but instead between those who had more challenging, direct experience with dementia and those who did not. It was those with negative direct experience who tended to have stronger favourable attitudes towards ADID and to rely more heavily on affective reasoning to substantiate their views. These individuals were primarily older adults who had directly cared for a parent or spouse, with one key exception, a younger adult who had also been in direct care of their parent and grandparent with dementia. Direct experience, particularly negative, has been shown to produce more certain and well-defined attitudes and our findings are consistent with this (Fazio & Zanna, 1978). The tripartite model of attitudes has been criticised for being unable to accurately predict behaviour (Kaiser & Wilson, 2019). With a behaviour as overt and irreversible as ADID, it is important we understand the true link between attitude and behaviour. For instance, research from the Netherlands found that terminally ill patients with positive attitudes towards assisted dying did not necessarily want an assisted death themselves (Johansen, Hølen, Kaasa, Loge, & Masterstvedt, 2005). This discrepancy is a key area for further investigation.

### Limitations

All participants in the study were of White ethnicity, the majority had some higher education and Christianity was the only religion represented in the sample. Research shows ethnicity, education and religion to be important factors associated with attitudes towards assisted dying (Tomlinson & Stott, 2015). The present findings may, therefore, have been different with the inclusion of a more diverse sample and must be interpreted with consideration of the specific demographic it represents. In addition, personal experience was more dominant in the sample than professional experience. The analysis was therefore more centred on personal experience and its relationship to attitudes towards ADID. A more focused sample might have given a richer understanding of one type of experience, although we believe the commonalities in attitudes from a range of different experiences makes the present research more widely applicable. Finally, participants’ responses may have been influenced by the group dynamic, particularly as the topic was of a sensitive and personal nature (Hollander, 2004). It is possible, therefore, that in-depth individual interviews may have garnered different results.

### Future Research

Future research should explore ADID with a broader range of participants, for instance, including individuals from a more diverse demographic, to gain insight into how these factors influence attitudes. Studies could also bring together different stakeholders including the individual with dementia, their family and their healthcare providers to explore how relational autonomy could be used to support decision-making and optimal care for individuals with dementia and their families.

## Conclusions

This paper is the first to compare the attitudes of younger and older adults in regard to ADID. Though at times differing in strength, the attitudes and the underlying rationale behind attitudes were markedly similar across the age groups. In light of the changing social landscape we face in the coming years, it is more important than ever to have frank and open discussions about how and when we want to die. For individuals with dementia and their families, focus should be on improving end-of-life care in dementia, including creating more options at the end of life, perhaps in the form of legalising ADID.
